# Nervous Diseases

**Published:** 1878-08

**Authors:** 


Nervous Diseases: their Description and Treatment. By Allan McLane Hamilton, Fellow of the New York Academy of Medicine, one of the attending physicians at the Epileptic and Paralytic Hospital, Blackwell's Island, New York City, etc., etc., with fifty-three illustrations. Philadelphia: Henry C. Lea. 1878.

Nervous diseases, the most difficult of all others to understand properly, are treated of in this work on the style of older authors. The various organic lesions producing nervous disturbance are not, we think, made sufficiently prominent for a practical treatise on the subject of nervous affections. Primary nervous diseases, however, are described in a manner to make the book interesting and instructive to the general practitioner.

